# Impact of COVID-19 on orthopedic surgery: Experience from Saudi Arabia

**DOI:** 10.1016/j.amsu.2020.05.048

**Published:** 2020-06-11

**Authors:** Ali H. Alyami, Alyazeed A. Alyami, Bandar N. AlMaeen

**Affiliations:** a Department of Surgery, Ministry of the National Guard - Health Affairs, Jeddah, Saudi Arabia; b King Saud Bin Abdulaziz University for Health Sciences, Jeddah, Saudi Arabia; c College of Medicine, King Abdulaziz University, Jeddah, Saudi Arabia; d Department of Surgery, College of Medicine, Jouf University, Sakaka, Saudi Arabia

**Keywords:** COVID-19, Orthopedic surgery, Saudi Arabia

## 1 Introduction

In December 2019, cases of pneumonia of unknown cause were detected in the city of Wuhan in the Hubei Province of China, and a report by the Chinese authorities to the World Health Organization (WHO Country Office) in China identified a new virus, severe acute respiratory syndrome coronavirus 2 (SARS-CoV-2), to be the cause of these cases [1,2]. The outbreak was declared a public health emergency of international concern in late January 2020 [3,4]. On February 11,2020, the disease caused by SARS-CoV-2 was officially named 2019 novel coronavirus (COVID-19) by the WHO [5].

On March 2, 2020, Saudi Arabia announced its first case of COVID-19 when its Ministry of Health detected the disease in a Saudi national returning from Iran via Bahrain [6]. As of May 9, 2020, there have been more than 4,010,537 confirmed cases worldwide and at least 275,960 deaths involving more than 215 countries, areas, or territories. As of the same date in Saudi Arabia, there have been 35,432 confirmed cases, of whom 229 have died and 9120 have recovered, giving a case fatality rate of 0.6% and a recovery rate of 23.1% [7].

The authors of a study published on May12, 2020, estimated that a total of 28,404,603 elective operations would be canceled or postponed worldwide during 12 weeks of disruption due to COVID-19 and ata rate of 2,367,050 operations per week [8]. Globally, their best estimate of the orthopedic surgery cancellation rate was approximately 82.0%, representing 6,295,041 of 7,677,515 cases.They concluded that if countries increased their normal surgical volumes by 20% after the pandemic, it would take about 45 weeks to clear the backlog of operations resulting from the disruption caused by COVID-19. It was estimated that the duration of COVID-19-related disruption would be about 11 weeks in Saudi Arabia.

The Saudi government, guided by the recommendations of its Ministry of Health, took aggressive measures to control the spread of COVID-19, implementing internal and external lockdowns in the early stage of the pandemic, which has reduced the rate of spread and resulted in less burden on the health care system. However, as in rest of the world, the COVID-19 pandemic has affected health care at the population level in our region.

## 2 Orthopedic surgery during the COVID-19 period

In the first two weeks of March 2020, the number of confirmed cases of COVID-19 in Saudi Arabia was small and all elective surgeries could proceed. However, with the increase in the number of cases in the second two weeks of March, the Ministry of Health advised that all elective surgeries in the public health sector should be put on hold. Exemptions were made at our institution for oncology cases and emergencies. Surgical practice has continued as normal in our private sector but with a reduction in the number of operations performed.

The rationale for deferring elective surgeries was to (1) increase the ability of hospitals to accommodate patients with COVID-19 in the event that a crisis point was reached and (2) limit the throughput of traffic within health care institutions to reduce the risk of cross-infection among health care workers, which was a major concern for all stakeholders.The bed capacity in our intensive care units is crucial at this time and stopping elective surgeries will reduce the occupancy rate.

We used the COVID-19 elective case triage guidelines for surgical care published by the American College of Surgeons as a reference to prioritize our surgical patients. Subsequently, all elective cases, including surgery in patients with cancer, were put on hold because of confirmed and suspected cases of COVID-19 among surgeons, anesthesiologists, nurses, and technicians, which led to the isolation of a large number of operating room staff. Fortunately, in most instances, tests for COVID-19 were negative and these staff members were able to return to work after being cleared by the infection prevention and control department. Thereafter, oncology surgery was resumed; however, only two operating rooms have been in use to preserve more functioning theater teams in the event of further cross-infection among theater staff.

Orthopedic surgery has been continued for oncology and emergency cases during the COVID-19 pandemic. Saudi Arabia has high rates of polytrauma and fractures related to motor vehicle accidents. However, these rates have been low during the COVID-19 pandemic, probably because of the lockdown. In Saudi Arabia, most cities went into partial lockdown that progressed to full lockdown for a few weeks and then subsequently returned to a partial lockdown again.

No routine preoperative testing for COVID-19 has been performed, likely for logistic reasons. As part of the Best Care system (an electronic medical record system), an acute respiratory illness screening tool has been applied for all hospital admissions, including all patients scheduled for surgery, to detect potential cases. Patient escorts are also screened. If the score is high, the patient or escort is swabbed and tested before surgery. Measures have also been taken in the operating room to deal with confirmed cases, including use of a negative pressure room, wearing of full personal protective equipment to protect staff, reducing traffic throughput, and removing all but essential staff members from the room during aerosol-producing procedures (e.g., during intubation).

A hospital scientific committee was created to review the emerging reports and research, to validate the evidence, and to provide all clinical departments with evidence-based recommendations for COVID-19 management. The third version of their recommendations included criteria for preoperative testing of surgical patients. Late in May 2020, preoperative testing for COVID-19 became routine for all surgical patients, except for urgent level 1cases, (i.e. where life-saving or limb-preserving surgery was required) which are treated as COVID-19 positive during surgery prior to receiving test results.

Delays in elective orthopedic surgery would have an impact on patient care, especially given the uncertainty regarding the duration of the COVID-19 pandemic. For example, the clinical scenario may worsen overtime in patients who need surgery for a spinal condition, arthroplasty revision, a benign tumor, or pediatric pathology.Therefore, we started to think about ade-escalation plan that could be synchronized with the strategy being implemented nationwide.

## 3 Impact on orthopedic outpatient clinics

Adaptation of our orthopedic outpatient clinics to accommodate the COVID-19 outbreak has passed through different phases. In the initial phase, the clinics were running regularly. With the escalation of preventive measures, those with the role of Most Responsible Physician were asked to review the list of scheduled patients and identify which patients should be seen in person according to their clinical status and which ones could be rescheduled. Active orthopedic oncology patients were exempted, as were new patients. The next phase saw the start of virtual clinics.

In orthopedics, patients are now notified by SMS message via a call center 48 h before hand to prepare themselves for a telephone call. If the patient's status mandates a physical assessment, the team arranges an attendance at the hospital, where all members of the orthopedic team and the patients wear surgical masks and gloves. Medications can be prescribed remotely and delivered to patients without the need to visit the pharmacy. Health care workers, patients, and escorts undergo a temperature check upon entering the main hospital or the ambulatory care center, where antiseptic preparations and sanitizers are available in all areas. Although virtual encounters have been a helpful solution, they may not be an optimal model and some patients may be affected by these changes.

## 4 Impact of COVID-19 on the residency training program

Unfortunately, our orthopedic residency training has been adversely affected by the COVID-19 pandemic. The inevitable pausing of all elective surgeries has limited hands-on practice and exposure to a number of procedures. Although the orthopedic oncology service has continued, this is a limited area within the orthopedic training program, which includes at least nine subspecialties. Trauma surgery has continued but at a much slower rate, most likely as a result of the nationwide lockdown. Running the outpatient clinics in a virtual manner has also reduced the opportunities for residents to perform physical examinations and manage their patients face-to-face. The weekly half-day reserved for academic activity has been converted to twice-weekly virtual learning. The Saudi Orthopedic Association has participated in all virtual learning activities by arranging webinars in the different subspecialties. The Saudi Commission for Health Specialties (SCFHS) has done the same but with more focus on COVID-19-related topics.

The SCFHS has asked the program's scientific committee to make recommendations regarding how the SCFHS should handle the residency programs during this critical period. The orthopedic scientific committee has recommended counting the 3-month rotation in the second quarter of 2020 as a general orthopedic rotation for junior residents and as an elective rotation for senior residents. If the pandemic continues, further recommendations will be made. For the time being, dozens of teams have created at our center as part of the staffing plan to cope with a crisis if one eventuates. The residents are part of these teams, including in orthopedics, but will not be involved unless we reach crisis mode.

## 5 Activities and education during the COVID-19 outbreak

In our country, the measures implemented by the Saudi government to protect all individuals in the community from COVID-19 infection have resulted in a sudden unexpected pause of all elective surgeries. One of the consequences of this is that surgeons, particularly those in the orthopedics field, are spending less time in operating rooms and have excellent opportunities for online learning during the lockdown period.

The Saudi Orthopedic Association and its subspecialty divisions, in cooperation with the Chapter Academy e-learning platform, have created a series of online interactive scientific lectures on upper extremity, sports, arthroplasty, oncology, deformity, foot and ankle, and other disciplines presented by a group of well-known and respected surgeons from the association and targeting practicing surgeons and trainees in the field.These live webinars are provided free of charge and have been successful in supporting continuing education and promoting an exchange of scientific ideas among orthopedic surgeons from all corners of the Kingdom of Saudi Arabia.

The Saudi Spine Society scientific committee has also released guidelines on how to increase awareness and exchange up-to-date information for health care providers regarding categorization of surgical spine procedures, limitations on postoperative physiotherapy programs, and management of all elective non-surgical interventions, as well as how not to interfere with the Ministry of Health's general guidelines for managing patients with infectious diseases, including COVID-19. The Saudi Spine Society and its regional branches have further maintained their regular spine club meetings via live webinars with no registration fees.

In some medical centers across the country, virtual education (e-learning) was started immediately in response to the COVID-19 pandemic. Orthopedic residents in some medical centers, including ours, are able to join in weekly case discussions using online meeting platforms presented by the departmental consultants. Many orthopedic surgeons in our country have also created educational social media videos in the Arabic language to reinforce the need for the Saudi population to stay in quarantine, to educate people about the most common reasons for an orthopedic clinic visit, and how to avoid unnecessary orthopedic injuries in the home.

## 6 Conclusions

The most important lesson we have learned while managing the COVID-19 outbreak has been the importance of effective communication and early intervention with plans for alternative ways to maintain the learning process along with the exchange of scientific ideas and education.We believe that the plans and actions taken during the COVID-19 outbreak in our country have been very effective in the prevention of further harm and afforded many lessons that can be applied to any similar crisis in the future.

## Provenance and peer review

Not commissioned, editor reviewed.

## Ethical approval

Not applicable.

## Author contribution

Ali H Alyami: concept and design, writing the paper, validation.

Alyazeed A Alyami: writing the paper.

Bandar N AlMaeen: concept and design, writing the paper.

## Sources of funding

The authors received no external funding for this research.

## Consent

Not applicable.

## Guarantor

Dr.Bandar N AlMaeen.

## Declaration of competing interest

On behalf of all authors, the corresponding author states that there is no conflict of interest.
